# Increased high-density lipoprotein cholesterol in patients with type 2 diabetes and its correlates: a cross-sectional, matched case–control survey

**DOI:** 10.1186/s40001-024-01950-0

**Published:** 2024-07-02

**Authors:** Fatemeh Heydarzadeh, Fatemeh Mohammadi, Amirhossein Yadegar, Ali Mohammadi Naeini, Seyed Ali Nabipoorashrafi, Soghra Rabizadeh, Alireza Esteghamati, Manouchehr Nakhjavani

**Affiliations:** Endocrinology and Metabolism Research Center (EMRC), Vali-Asr Hospital, Tehran University of Medical Sciences, Tehran, Iran

**Keywords:** Cholesterol, Triglyceride, Vitamin D, Lipoproteins, Lipids, High-density lipoprotein

## Abstract

**Background:**

So far, high-density lipoprotein cholesterol (HDL-C) levels and mortality were shown to have a U-shaped relationship. Additionally, high HDL-C levels increase the risk of developing a variety of diseases. However, a paucity of data exists regarding the characteristics of people with high HDL-C levels. The aim of this study was to assess the demographics and characteristics of patients with high HDL-C levels and compare their features with normal and low HDL-C groups.

**Methods:**

As a cross-sectional, matched case–control study, a total of 510 patients with type 2 diabetes (T2D) were enrolled in the study and categorized into three matched groups according to their HDL-C concentrations. The studied groups were matched by their age and gender. Restricted cubic spline (RCS) curves were designed to evaluate the relationship between height, blood pressure, triglyceride, and vitamin D concentrations with the probability of having high HDL-C levels. Furthermore, violin plots were conducted to illustrate the distribution of continuous variables within each group.

**Results:**

This study showed that having high HDL-C (more than 70 mg/dL) compared to having low HDL-C (less than 40 mg/dL in men and 50 mg/dL in women) was significantly associated with height (OR 0.918, 95% CI 0.866–0.974), systolic blood pressure (SBP) (0.941, 0.910–0.972), vitamin D (0.970, 0.941–0.999), and triglyceride (0.992, 0.987–0.998) serum concentrations. Further analysis investigated that having high HDL-C levels compared to desired HDL-C levels (40 ≤ HDL-C levels < 70 in men and 50 ≤ HDL-C levels < 70 in women) was inversely associated with having SPB values greater than 130 mmHg. Besides, sufficient vitamin D levels (above 20 ng/ml) could 0.349 times decrease the odds of having high HDL-C versus normal HDL-C levels.

**Conclusion:**

Sufficient vitamin D levels, SPB values higher than 130 mmHg, as well as increased triglyceride levels, were inversely associated with having high HDL levels. However, higher height values were associated with a decreased likelihood of having high HDL.

## Introduction

High-density lipoprotein cholesterol (HDL-C) is a small lipoprotein with a complex structure determined by various particle sizes [1]. The prevalence of reduced HDL-C levels in the general population, less than 40 in men and 50 in women, has been reported to be 41.1% in Africa, followed by 35.4% in Europe, 33.4% in Korea, 21.1% in Turkey, 19.2% in China, 12.4%-33.1% in the United States, and 42% in Iran [2–4]. Literature has confirmed that low concentrations of HDL-C are associated with infectious diseases, sepsis-related death, diabetes, chronic kidney disease, various autoimmune diseases, and ruptured intracranial aneurysms [5, 6].

Furthermore, the prevalence of high HDL-C levels greater than 70, was 26.6% among selected women in Tunisia [7]. High HDL-C levels were also reported in more than a third of type 1 diabetes (T1D) cases [8]. Potential regulatory roles are pictured for HDL-C [1, 9]. HDL-C can directly regulate glucose metabolism and results in its antidiabetic effects. Current evidence has suggested that high HDL-C concentrations are risk factors for infectious diseases, pterygium, end-stage renal disease (ESRD) in lupus nephritis, increased insulin resistance in metabolic syndrome, and all-cause mortality [5, 10–13]. Accordingly, a U-shaped relationship between HDL-C levels and cardiovascular and cancer mortality has been plotted [14].

Recent evidence supports the idea that high HDL-C levels (>50 mg/dL) can increase the cancer mortality rate [7]. The antioxidant effects of HDL-C have been manifested in prostate, lung, and endometrial cancer [15]. However, breast cancer could be promoted by HDL-C through increased migration of cancer cells. A positive correlation between high HDL-C and cancer progression was also demonstrated regarding the role of scavenger receptor BI proteins (SRB-1) in facilitating HDL-mediated cholesterol ester absorption by tumor cells [16]. Low HDL-C levels were also suggested to be endangered for cancer-related death [17].

Earlier, an inverse linear association was described between HDL-C levels, cardiovascular events, and all-cause mortality. Atherosclerosis development could be protected through different HDL-C capacities, including cholesterol removal from the artery wall, vasodilation in endothelial cells, protective effects on low-density lipoprotein (LDL) oxidation, and anti-inflammatory effects[9, 18]. To date, there is a lack of documented conclusive evidence for the effectiveness of HDL-C-raising drugs [9, 19, 20]. Although it has been shown that niacin and cholesterol ester transfer protein (CETP) inhibitors could lead to an increase in HDL-C levels, the cardiovascular risk was not substantially reduced using such medications [5]. However, the cardioprotective role of high HDL-C has been questioned recently[14]. Recently, the Multi-Ethnic Study of Atherosclerosis (MESA) hypothesized that there may be a link between high HDL-C levels (>60 mg/dL), and a greater incidence of myocardial scars and that high HDL-C levels are associated with greater interstitial fibrosis, which manifests as longer myocardial native T1 times and greater extracellular volume [21]. Chronic kidney disease (CKD), diabetes, and coronary artery disease can cause dysfunctional HDL-C production, altering its anti-inflammatory properties [22].

So far, several pieces of research have been conducted on the effects of low HDL levels on cancer, cardiovascular events, and diabetes [16, 23–25]. Recent studies have reported failure in reducing cardiovascular events, insulin resistance, diabetic retinopathy, cancer mortality rate, and unexpectedly increasing mortality in subjects with elevated HDL‐C levels [7, 13, 14, 26]. However, there is a paucity of data on the characteristics of those with high HDL-C levels. On the other hand, there is no definition of the maximum level of normal HDL-C so far. The present survey tried to shed light on other correlates of high HDL-C. Studying the demographic and laboratory findings of individuals with high HDL-C levels could further increase the insights of the scientists toward the elements that could be effective in low or high HDL management. This study tried to examine all the demographics and characteristics of patients with low, normal, and high HDL-C levels in three age- and gender-matched groups.

## Materials and methods

### Study design and population

A total of 7391 consecutive patients with type 2 diabetes (T2D) who were referred to a university hospital affiliated with the Tehran University between 2016 and 2021 were retrospectively recruited. Individuals who were unwilling to participate in the study; who were pregnant; who used aspirin, oral contraceptive drugs, or antioxidant and vitamin supplements; who were not receiving statins; who had several other chronic conditions (i.e., thyroid dysfunction, history of liver cirrhosis, CKD, or cancer); who had a history of smoking; or who were consuming alcohol were excluded from the study. As a result, 6127 patients were enrolled in the study. In the matching process, age and sex were determined as matching variables. Subjects with low, normal, and high HDL levels were matched for age and sex. According to the power of the study as 0.95 and ‘α’ as 0.05, utilizing G*Power software version 3.1.9.2 (Universität Düsseldorf, Germany), a total number of 400 was calculated [27]. Therefore, in the study period, 510 participants with T2D were included. A total of 170 patients with low HDL-C, 170 patients with normal HDL-C, and 170 patients with high HDL-C were matched by sex and age.

A high ratio of the target population had middle socioeconomic status, most of whom were covered by insurance. Patients were taking oral antidiabetic drugs (OADs), insulin, or a combination of these drugs. Informed written consent was obtained from all study populations. This study was in accordance with the Declaration of Helsinki. The study received formal ethical approval from the local ethics committee of Tehran University of Medical Sciences.

### Data collection

The baseline characteristics of the recruited participants, including general information (age, sex, body mass index (BMI), hypertension status, hyperlipidemia status, diabetes duration, height, weight, waist circumference, and hip circumference) and laboratory test results (LDL-C, HDL-C, triglyceride (TG), total cholesterol (TC), non-HDL-C, fasting blood glucose (FBG), two-hour postprandial glucose (2hPP), hemoglobin A1c (HbA1c), vitamin D, creatinine and urinary albumin) were recorded.

A portable stadiometer and calibrated balance beam scale were employed to determine height and weight, respectively. WC was assessed halfway between the lowest rib margin and the iliac crest. Body mass index (BMI, kg/m^2^) was calculated by using weight (kg)/height ^2^ (m ^2^). BMI was classified into three categories: underweight (BMI<18.5 kg/m^2^), normal (18.5 kg/m^2^ ≤ BMI < 25 kg/m^2^), and overweight/obese (BMI ≥ 25 kg/m^2^). The cut-off values for waist circumference (WC) were considered 98 cm for males and 84 cm for females [28, 29]. Blood pressure was recorded in the right arm in the sitting position after the participants had rested for 5 mins. The individual’s right arm was placed at heart level and blood pressure was measured with a calibrated mercury sphygmomanometer (Reishter, Germany). The recorded data included the average of the last two systolic and diastolic pressures. All measurements were accomplished with an accuracy of 0.1 cm. After 12 h of overnight fasting, blood samples were collected in tubes coated with ethylene diaminetetracetic acid. All the samples were kept on ice and centrifuged at 3000 rpm for 15 min at 4 °C. Cholesterol, HDL, LDL, and TG were measured using direct enzymatic colorimetry with a Technicon RA-analyzer (Pars Azmoon, Karaj, Iran). Fasting blood sugar (FBS) and two-hour postprandial glucose (2 hPP) were quantified via the glucose oxidase test. Glucose level was assessed by the glucose oxidase method with an intra-assay coefficient of variation (intra-assay CV = 2.1%; interassay CV = 2.6%). High-performance liquid chromatography (HPLC) (A1C, DS5 Pink kit; Drew, Marseille, France) was utilized to evaluate hemoglobin A1c (HbA1c) levels. Non-HDL-C was estimated by reducing HDL-C from total cholesterol. The following equation (log (TG/HDL-C)) was performed to compute the atherogenic index of plasma (AIP). (WC(cm)/(39,68+(1.88*BMI) *(TG/1.03) *(1.31/HDL) for men and (WC(cm)/(36,58+(BMI *1.89) *(TG/0.81) *(1.52/HDL) for women were assessed to calculate the visceral adiposity index (VAI). eGFR was measured by using the Chronic Kidney Disease Epidemiology Collaboration (CKD-EPI) equation. Serum creatinine was measured using the Jaffe method (Pars Azmun, Karaj, Iran)

Urinary albumin concentrations were measured by immunoturbidimetry (Cecile Instruments, Cambridge, United Kingdom). The detection limit was established at 2 mg/L. Urinary albumin concentrations were evaluated by an immunoturbidimetric commercial kit (Randox, Antrim, UK) [30–32].

### Definitions

The criteria for defining diabetes mellitus followed the guidelines of the American Diabetes Association (ADA) [33]. Dyslipidemia was described according to the NCEP ATP III (National Cholesterol Education Program-Adult Treatment Panel III) and AHA/ACC (The American Heart Association/The American College of Cardiology) guidelines [34, 35]. Low HDL-C (< 40 mg/dL in men and < 50 mg/dL in women), high LDL-C (≥ 70 mg/dL), high total cholesterol (≥ 200 mg/dL), high non-HDL-C (≥ 130 mg/dL), high TG (≥ 150 mg/dL), and high AIP (> 0.24) were defined according to guidelines. Microalbuminuria was defined as the excretion of between 30 and 300 mg/day of urine albumin.

### Statistical analysis

**Fig. 1:**
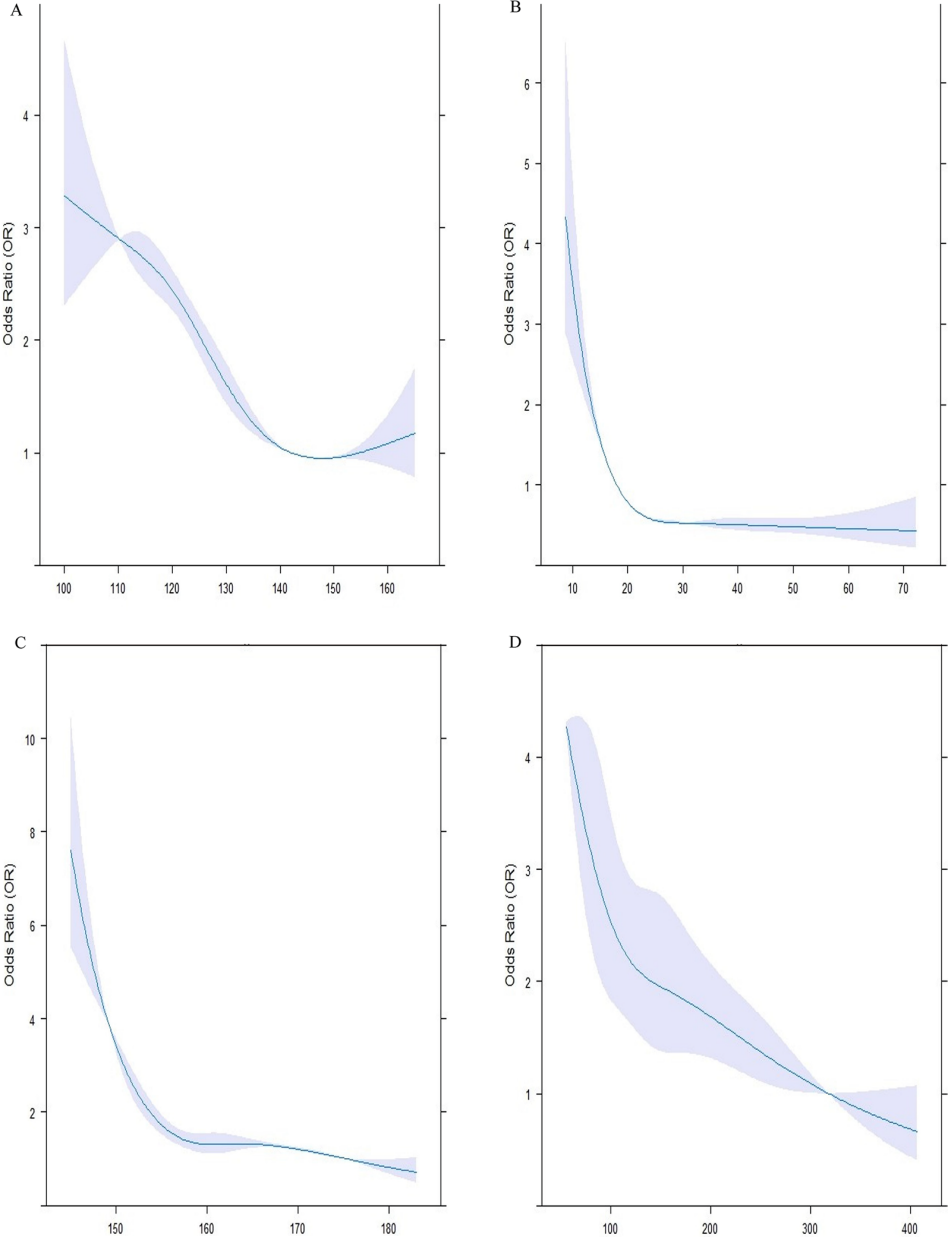
Association between systolic blood pressure (SBP), Vitamin D, height, and triglyceride and high HDL-C levels. The RCS models were used to analyze the relationship between SBP (**A**), vitamin D (**B**), height (**C**) and triglyceride (**D**) concentrations, and the probability of having HDL-C levels of more than 70. Each RCS consisted of four knots based on the distribution of the associated variable. The reference values for the abovementioned curves were as follows: height of 160 cm, SBP of 130 mm Hg, triglyceride levels of 150 mg/dl, and vitamin D levels of 20 mg/dl. HDL-C: high-density lipoprotein cholesterol

**Fig. 2:**
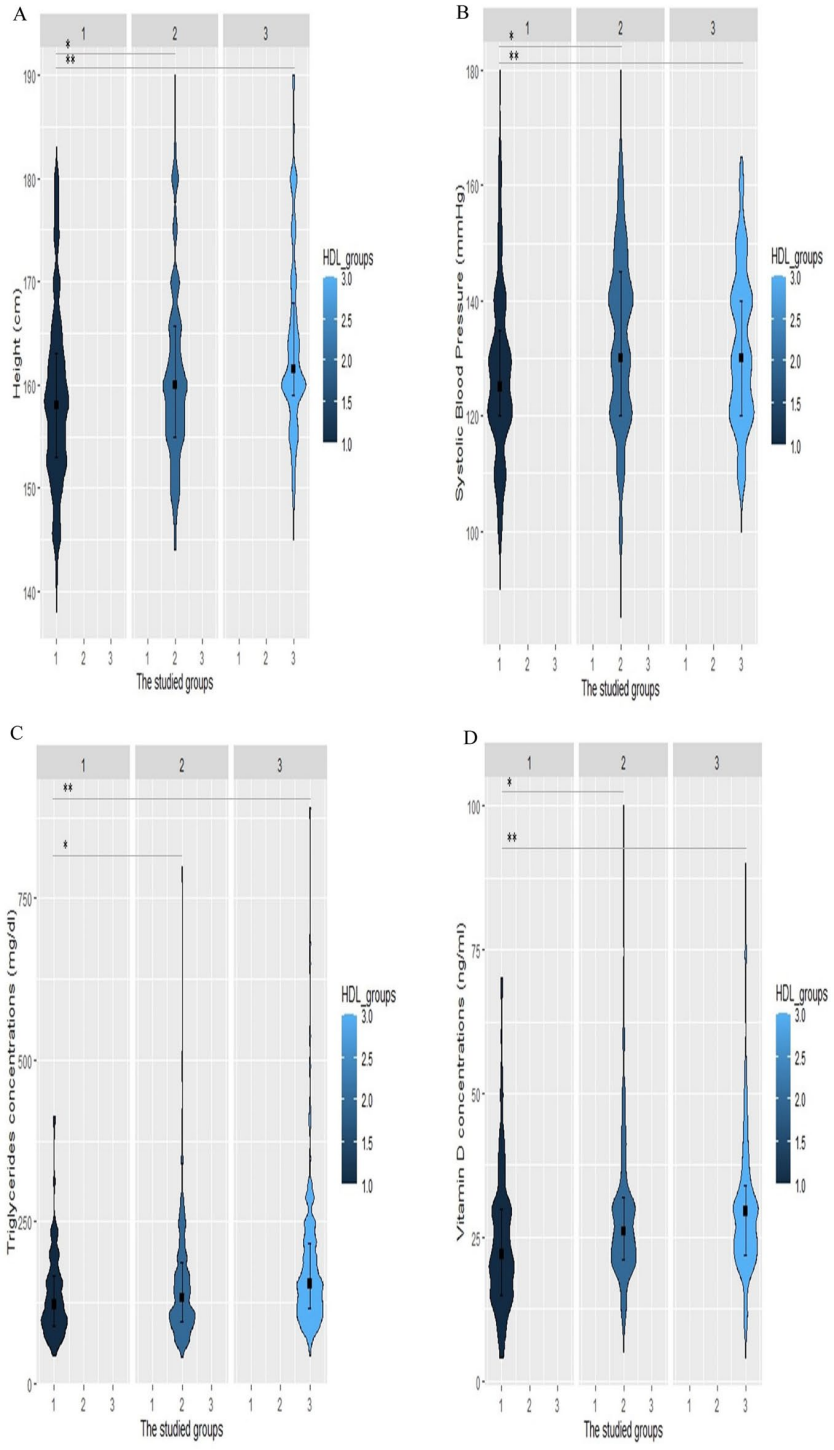
Violin plots of height, systolic blood pressure (SBP), triglyceride, and vitamin D levels for increased HDL-C levels. Violin plots were used to visually represent the density of height (**A**), SBP (**B**), triglyceride (**C**), and vitamin D (**D**) levels groups with high (70 ≤ HDL-C), normal HDL-C levels (40 ≤ HDL-C < 70 in men, 50 ≤ HDL-C < 70 in women), and low HDL-C levels (HDL-C <40 in men, HDL-C <50 in women). The median height was 161.5 cm, 160 cm, and 158 cm in the low HDL-C, normal HDL-C, and high HDL-C groups, respectively (Fig. 2A). The group with high HDL-C levels had significantly lower SBP with a median value of 125 mm Hg (Fig. 2B). Individuals with HDL-C levels of more than 70 had significantly lower levels of triglyceride [122 (IQR, 89–126)] and vitamin D [22 (IQR, 15–30)]. *p-value < 0.05 vs group with normal HDL, **p-value < 0.05 vs group with low HDL, HDL-C: high-density lipoprotein cholesterol

All the statistical analyses were carried out using R software (version 4.2.3, R Foundation for Statistical Computing, Vienna, Austria) and IBM SPSS software version 24.0 (SPSS Inc., Chicago, Illinois, USA). The normality of the data was assessed with the Shapiro–Wilk test. Normal distribution quantitative values were represented by the mean ± standard deviation (SD) such as age, whereas data with no normal distribution was displayed as the median [interquartile range (IQR)], including duration of diabetes (years), weight (kg), height (kg), BMI (kg/m^2^), WC (cm), hip circumference (cm), SBP (mm Hg), DBP (mm Hg), serum levels of vitamin D, total cholesterol (mg/dL), LDL-C (mg/dL), HDL-C (mg/dL), TG (mg/dL), non-HDL-C (mg/dL), AIP, TG/HDL ratio, HbA1c (%), FBS (mg/dL), 2 hPP (mg/dL), creatinine (mg/dL), GFR (ml/min/1.73 m^2^), and microalbuminuria (mg/12 h). IQR was defined as the range between the first and third quartiles. Categorical parameters were expressed in terms of percentages and numbers [ n (%)]. Of 6127 patients, 510 subjects were matched by age and gender and assigned to three groups according to their HDL-C levels. The first group consisted of patients with low HDL-C levels (HDL-C < 40 in men, HDL-C < 50 in women), the second group contained those with normal HDL-C levels (40 ≤ HDL-C < 70 in men, 50 ≤ HDL-C < 70 in women), and the third group had high HDL-C levels (70 ≤ HDL-C). Each group contained 170 age-and-gender-matched individuals. ANOVA and Kruskal-Wallis tests were applied along with their Post Hoc pairwise comparisons to evaluate the baseline features of patients with normal and non-normal distributions. Between-group comparisons were assessed using Chi-square. Binary conditional logistic regression was employed to assess odds ratios (OR) and their 95% confidence intervals (CI) of patient’s characteristics for having high HDL-C levels. Univariate and multivariate regression models were analyzed. Multivariate model 1 was adjusted for height, WC, SBP, Vitamin D, HBA1c, the status of hypertensive drugs, the anti-diabetic drugs, the type of lipid-lowering drug, duration of diabetes, creatinine and triglyceride concentration. Multivariate model 2 was adjusted for height, SBP, Vitamin D, the status of hypertensive drugs, the anti-diabetic drugs, the type of lipid-lowering drug, duration of diabetes, and triglyceride concentration after dividing into different categories. Restricted cubic spline (RCS) curves in Fig. 1 with 4 knots were further utilized to explore the relationships between height (Fig. 1C), SBP (Fig. 1A), triglyceride (Fig. 1D), and vitamin D (Fig. 1B) concentrations with the risk of having high HDL-C levels. Violin plots (Fig. 2) were also designed to reveal the distribution of continuous variables, which were significantly different among the three groups. A two-sided P-value of less than 0.05 was assumed to be the threshold for statistical significance.

## Results

### Baseline characteristics

In this study, 510 patients with T2D who were matched by their age and gender were stratified into three strata based on their HDL-C levels. The mean (± SD) age of the total participants was 59.83 ± 9.48 years. A majority of the population consisted of women, approximately 76.7%. The median duration of diabetes was six years (minimum five and maximum 38 years). The median BMI (kg/m^2^) was 27.99 (IQR, 25.00–31.23). About 42.4% of participants were receiving anti-hypertensive drugs. The median values of SBP and DBP (mmHg) were 130 and 80, with ranges of 85 to 190 and 50 to 103, respectively. Serum levels of vitamin D had a median of 25 (ng/ml)(IQR, 20.00–32.00). All individuals were receiving statins, including atorvastatin (81%) and rosuvastatin (19 %). The median concentrations of total cholesterol (mg/dL), LDL-C (mg/dL), and HDL-C (mg/dL) were 170 (IQR, 145.00–205.00), 90 (IQR, 68.00–115.00), and 53 (IQR, 42.00–72.00), respectively. The median TG level was 136 (IQR, 99.00–190.00). All participants were taking antidiabetic agents, including OAD (76.4%), insulin (14.7%), and their combination (8.7%). Despite diabetes medication use, the median of HbA1c was 7.50 (IQR, 6.50–8.80), followed by 145 (IQR, 120.00–187.00) and 199.5 (IQR, 150.00–256.50) for FBS (mg/dL) and 2hPP (mg/dL), respectively. Microalbuminuria (mg/12 h) was computed to have a median of 7.50, along with a range of 4 to 13.57, respectively. Retinopathy and neuropathy were diagnosed in about 6.1% and 12.2% of the studied population. A total of 12.5% of participants reported a history of cardiovascular disease (CVD) in their visits.

Detailed profiles of participants in three categories stratified by HDL-C levels (defined as low HDL-C levels (HDL-C < 40 in men, HDL-C < 50 in women), normal HDL-C levels (40 ≤ HDL-C < 70 in men, 50 ≤ HDL-C < 70 in women), and high HDL-C levels (70 ≤ HDL-C)) were also investigated. A notable discrepancy was recorded in the duration of diabetes, height, waist circumference, SBP, vitamin D levels, lipid profile and parameters, and microalbuminuria among the three groups. Weight and non-HDL-C levels were significantly different in the high HDL-C group versus the low HDL-C group. In addition, individuals in the high HDL-C category had substantially greater differences in BMI and hip circumference than did those in the normal HDL-C category (Table 1) All demographic, anthropometric, clinical, and laboratory details of individuals in each classification are presented in Table 1.

**Table 1:** Characteristics of the patients at baseline

Variable		Total (n = 510)	Low HDL (HDL-C < 40 in men, HDL-C < 50 in women) (n = 170)	Normal HDL (40 ≤ HDL-C < 70 in men, 50 ≤ HDL-C < 70 in women) (n = 170)	High HDL (70 ≤ HDL-C) (n = 170)
Age (yr)		59.83 ± 9.48	58.93 ± 10.27	60.92 ± 9.10	59.64 ± 8.97
Duration of diabetes (yr)		6.00(2.00–12.00)	5.00(2.00–10.00)	5.00(2.00–10.00)	8.50(3.00–14.00)^a,b^
Weight (kg)		73.00(65.75–82.00)	75.00(69.00–84.25)	70.50(64.75–80.00)	72.00(64.00–80.00)^a^
Height (cm)		160.00(155.00–166.00)	161.50(159.00–168.50)	160.00(155.00–166.00)	158.00(153.00–163.25)^a,b^
BMI (kg/m^2)		27.99(25.00–31.23)	28.10(25.55–31.15)	27.11(24.67–30.40)	28.83(24.97–32.03)^b^
BMI category	Underweight (< 18.5 kg/m^2^)	0.2%	0.0%	0.6%	0.0%
	Normal (18.5 kg/m^2^ ≤ BMI < 25 kg/m^2^)	24.7%	21.8%	27.1%	25.3%
	Overweight and Obese (≥ 25 kg/m^2^)	75.1%	78.2%	72.4%	74.7%
Presence of high waist circumference (cm)	Female (≥ 84 cm)	72.4%	72.9%	74.1%	70.0%
	Male (≥ 98 cm)	13.1%	16.4%	13.5%	9.4%
Waist circumference (cm)		100.00(92.00–103.00)	102.00(96.75–104.00)	99.00(92.00–103.00)	95.00(88.00–102.25)^a,b^
Hip circumference (cm)		105.00(102.00–109.00)	105.00(103.00–109.00)	104.00(100.00–108.00)	106.00(100.00–112.00)^b^
Blood pressure (mm Hg)	Systolic	130.00(120.00–140.00)	130.00(120.00–140.00)	130.00(120.00–145.00)	125.00(120.00–135.00)^a,b^
	Diastolic	80.00(70.00–80.00)	80.00(70.00–80.00)	80.00(70.00–80.00)	80.00(70.75–80.00)
Vitamin D (ng/mL)		25.00(20.00–32.00)	29.50(22.00–34.00)	26.00(21.00–32.00)	22.00(15.00–30.00)^a,b^
HbA1c (%)		7.50(6.50–8.80)	7.50(6.60–8.62)	7.30(6.50–8.62)	7.50(6.40–9.00)
FBS (mg/dL)		145.00(120.00–187.00)	146.50(119.75–183.00)	140.50(120.00–181.25)	146.50(120.00–195.75)
2hPP(mg/dL)		199.50(150.00–256.50)	195.00(150.00–255.00)	199.00(152.50–240.00)	200.50(145.00–288.50)
Cholesterol (mg/dL)	Total	170.00(145.00–205.00)	150.00(124.75–177.00)	166.50(146.00–199.00)	200.00(170.75–236.25) ^a,b^
	LDL-C	90.00(68.00–115.00)	80.00(62.75–102.25)	90.00(69.00–110.50)	100.50(79.00–130.25) ^a,b^
	HDL-C	53.00(42.00–72.00)	38.50(35.00–44.00)	53.00(50.00–59.00)	74.00(72.00–81.00) ^a,b^
Triglyceride (mg/dL)		136.00(99.00–190.00)	153.50(115.50–216.00)	132.00(95.75–188.00)	122.00(89.00–166.00) ^a, b^
Non-HDL-C (mg/dL)		114.00(90.00–145.00)	109.00(87.75–136.00)	112.50(93.75–143.00)	121.50(94.00–158.50) ^a^
AIP		0.91(0.48–1.36)	1.41(1.00–1.73)	0.91(0.55–1.26)	0.48(0.15–0.78) ^a,b^
TG/HDL ratio		2.48(1.62–3.93)	4.13(2.72–5.68)	2.48(1.74–3.54)	1.61(1.17–2.19) ^a,b^
Creatinine (mg/dL)		0.90(0.80–1.10)	0.90(0.80–1.10)	0.90(0.80–1.02)	0.90(0.80–1.00)
Microalbuminuria (mg/12h)		7.50(4.00–13.57)	5.00(4.00–12.00)	6.00(3.00–11.00)	10.00(5.00–19.00)^a,b^
GFR (ml/min/1.73 m^2^)		77.76(61.65–95.25)	81.60(64.23–100.51)	75.13(59.49–95.70)	77.54(62.27–90.27)
Presence of Retinopathy		6.1%	3.5%	3.2%	11.8%*
Presence of Neuropathy		12.2%	10.1%	6.5%	22.5%*
Presence of HTN		39.2%	38.8%	45.9%	32.9%
History of CAD		12.5%	12.4%	13.5%	11.8%

Data are presented as means ± SD, median (interquartile range: first quartile- third quartile), or n (%)

BMI: body mass index; HbA1c: hemoglobin A1C; FBS: fasting blood sugar; 2hPP: two-hour postprandial glucose; LDL-C: low-density lipoprotein cholesterol; HDL-C: high-density lipoprotein cholesterol; TG: triglyceride; GFR: glomerular filtration rate; AIP: Atherogenic index of plasma*;* HTN: hypertension; CAD: coronary artery disease

^a^p-value < 0.05 high HDL vs low HDL derived from ANOVA or Kruskal–Wallis test

^b^p-value < 0.05 high HDL vs normal HDL derived from ANOVA or Kruskal–Wallis test

^*^p-value < 0.05 in the χ^2^ test

To visualize the smoothed density of height, SBP, triglyceride, and vitamin D among patients with high(70 ≤ HDL-C), normal(40 ≤ HDL-C < 70 in men, 50 ≤ HDL-C < 70 in women), and low(HDL-C < 40 in men, HDL-C < 50 in women) HDL-C levels, violin plots were constructed. Violin plot results revealed that the median height was 161.5 cm, 160 cm, and 158 cm in the low HDL-C, normal HDL-C, and high HDL-C groups, respectively (Fig. 2A). As shown in Fig. 2B the median SBP was 125 mmHg and the group with high HDL-C levels had significantly lower SBP values. Plots for triglyceride (Fig. 2C) and vitamin D (Fig. 2D) concentrations followed a similar pattern, which demonstrated that those with HDL-C levels greater than 70 had considerably lower levels of triglyceride [122 (IQR, 89–126)] and vitamin D [22(IQR, 15–30)].

### Associations of the studied values with high HDL-C concentrations and low HDL-C concentrations

According to the crude models of conditional logistic regression analysis, there was a substantial negative association between HDL-C levels and height, weight, SBP, vitamin D, and triglycerides. In addition, after categorizing the variables, which had a significant association with having high HDL levels, into two groups according to their reference points in the RCS, their OR remained significant (Table 2) No considerable associations were illustrated between having a normal HDL-C level and having other HDL-C levels.

**Table 2:** Odds of having high HDL-C levels (70 mg/dL ≤ HDL-C) compared to having low HDL-C levels (< 40 mg/dL in men and < 50 mg/dL in women) in the studied population with type 2 diabetes

Univariate	OR		95% CI	p-value
Height	0.936		0.913–0.962	< 0.001
Height in category	Height < 160 cm	1.00 (reference)		
	Height ≥ 160 cm	0.251	0.159–0.397	< 0.001
Weight	0.973		0.956–0.991	0.003
BMI	1.013		0.967–1.060	0.595
Waist circumference	0.936		0.912–0.962	< 0.001
Hip circumference	1.007		0.974–1.040	0.679
SBP	0.977		0.962–0.992	0.002
SBP in category	SBP < 130 mmHg	1.00 (reference)		
	SBP ≥ 130 mmHg	0.566	0.368–0.870	0.009
DBP	1.036		1.009–1.064	0.007
Vitamin D	0.962		0.939–0.986	0.002
Vitamin D in category	Vitamin D < 30 ng/mL	1.00 (reference)		
	Vitamin D ≥ 30 ng/mL	0.469	0.275–0.799	0.005
Vitamin D in category	Vitamin D < 20 ng/mL	1.00 (reference)		
	Vitamin D ≥ 20 ng/mL	0.159	0.079–0.317	< 0.001
FBS	1.003		0.999–1.006	0.138
2hpp	1.002		0.999–1.004	0.125
HbA1c	1.108		0.975–1.258	0.115
Triglyceride	0.993		0.989–0.996	< 0.001
Triglyceride in category	Triglyceride < 150 mg/dL	1.00 (reference)		
	Triglyceride ≥ 150 mg/dL	0.484	0.311–0.754	0.001
Creatinine	0.536		0.197–1.454	0.221

OR: odds ratio, 95% CI: 95% confidence interval

BMI: body mass index; SBP: systolic blood pressure; DBP: diastolic blood pressure; HbA1c: hemoglobin A1C; 2hpp: two-hour postprandial glucose; FBS: fasting blood sugar

The variables are being divided into two groups based on their reference points in RCS

After further adjustments in the multivariable model, having high HDL-C levels compared to having low HDL-C levels remained significantly associated with height (OR 0.918, 95% CI 0.866–0.974), SBP (0.941, 0.910–0.972), vitamin D (0.970, 0.941–0.999), and triglyceride (0.992, 0.987–0.998) serum concentrations. After controlling for other variables, patients with heights less than 160 cm, SBP below 130 mm Hg, Vitamin D levels of less than 30 ng/ml, and triglyceride concentrations of less than 150 mg/dL were more susceptible to having HDL-C levels greater than 70 than to having low HDL-C levels (Table 3)

**Table 3:** Odds of having high HDL-C levels (70 mg/dL ≤ HDL-C) compared to having low HDL-C (< 40 mg/dL in men and < 50 mg/dL in women) in the studied population with type 2 diabetes according to the multivariate models

Multivariate		OR	95% CI	p-value
Model 1^a^				
Height		0.918	0.866–0.974	0.004
SBP		0.941	0.910–0.972	< 0.001
Vitamin D		0.970	0.941–0.999	0.047
Triglyceride		0.992	0.987–0.998	0.008
Model 2^b^				
Height in category	Height < 160 cm	1.00 (reference)		
	Height ≥ 160 cm	0.277	0.127–0.607	0.001
SBP in category	SBP < 130 mmHg	1.00 (reference)		
	SBP ≥ 130 mmHg	0.387	0.190–0.789	0.009
Vitamin D in category	Vitamin D < 30 ng/mL	1.00 (reference)		
	Vitamin D ≥ 30 ng/mL	0.422	0.205–0.869	0.019
Triglyceride in category	Triglyceride < 150 mg/dL	1.00 (reference)		
	Triglyceride ≥ 150 mg/dL	0.352	0.153–0.807	0.013

OR: odds ratio, 95% CI:  95% confidence interval

SBP: systolic blood pressure, HDL-C: high density lipoprotein cholesterol

^a^Model 1 was adjusted for height, waist circumference, SBP, Vitamin D, HBA1c, the status of hypertensive drugs, the anti-diabetic drugs, the type of lipid-lowering drug, duration of diabetes, creatinine, and triglyceride concentration

^b^Model 2 was adjusted for height, SBP, Vitamin D, the status of hypertensive drugs, the anti-diabetic drugs, the type of lipid-lowering drug, duration of diabetes, and triglyceride concentration after dividing into different categories

### Associations of the studied values with high HDL-C concentrations and normal HDL-C concentrations

In the unadjusted analysis, substantial relationships were detected between high HDL-C and height (0.971, 0.948–0.995), waist circumference (0.9971, 0.948–0.995), SBP (0.974, 0.960–0.988), and Vitamin D level (0.969, 0.947–0.991) (Table 4). However, after adjustments for confounding variables, only height, SBP, and vitamin D concentrations remained significantly associated with having high HDL-C compared to having normal HDL-C levels. Further analysis revealed that a diagnosis of SPB greater than 130 mm Hg could be inversely associated with having HDL-C levels greater than 70 mm Hg compared to having the desired HDL-C values. In addition, sufficient vitamin D concentrations (above 20 ng/ml) could 0.349 times decrease the odds of having high HDL-C versus normal HDL-C levels (Table 5).

**Table 4:** Odds of having high HDL-C levels (70 mg/dL ≤ HDL-C) compared to having normal HDL-C(40 ≤ HDL-C < 70 in men, 50 ≤ HDL-C < 70 in women) in the studied population with type 2 diabetes

Univariate	OR		95% CI	p-value
Height	0.971		0.948–0.995	0.021
Height in category	Height < 160 cm	1.00 (reference)		
	Height ≥ 160 cm	0.608	0.396–0.934	0.023
Weight	1.002		0.985–1.020	0.795
BMI	1.042		0.995–1.090	0.078
Waist circumference	0.971		0.948–0.995	0.018
Hip circumference	1.026		0.995–1.058	0.095
SBP	0.974		0.960–0.988	< 0.001
SBP in category	SBP < 130 mmHg	1.00 (reference)		
	SBP ≥ 130 mmHg	0.463	0.300–0.716	< 0.001
DBP	1.021		0.995–1.047	0.111
Vitamin D	0.969		0.947–0.991	0.007
Vitamin D in category	Vitamin D < 30 ng/mL	1.00 (reference)		
	Vitamin D ≥ 30 ng/mL	0.600	0.350–1.029	0.063
Vitamin D in category	Vitamin D < 20 ng/mL	1.00 (reference)		
	Vitamin D ≥ 20 ng/mL	0.235	0.123–0.445	< 0.001
FBS	1.003		0.999–1.007	0.062
2hpp	1.002		0.999–1.004	0.090
HbA1c	1.083		0.960–1.221	0.194
Triglyceride	0.997		0.994–1.000	0.080
Triglyceride in category	Triglyceride < 150 mg/dL	1.00 (reference)		
	Triglyceride ≥ 150 mg/dL	0.768	0.493–1.196	0.243
Creatinine	0.987		0.357–2.733	0.981

OR: odds ratio, 95% CI: 95% confidence interval

BMI: body mass index; SBP: systolic blood pressure; DBP: diastolic blood pressure; HbA1c: hemoglobin A1c; 2hPP: two-hour postprandial glucose; FBS: fasting blood sugar

**Table 5:** Odds of having high HDL-C levels (70 mg/dL ≤ HDL-C) compared to having normal HDL-C(40 ≤ HDL-C < 70 in men, 50 ≤ HDL-C < 70 in women) in the studied population with type 2 diabetes according to the multivariate models

Multivariate		OR	95% CI	p-value
Model 1^a^				
Height		0.950	0.902–0.999	0.047
SBP		0.965	0.943–0.988	0.003
Vitamin D		0.968	0.942–0.994	0.016
Model 2^b^				
SBP in category	SBP < 130 mmHg	1.00 (reference)		
	SBP ≥ 130 mmHg	0.478	0.251–0.909	0.024
Vitamin D in category	Vitamin D < 20 ng/mL	1.00 (reference)		
	Vitamin D ≥ 20 ng/mL	0.349	0.170–0.718	0.004

OR: odds ratio, 95% CI: 95% confidence interval

^a^Model 1 was adjusted for height, waist circumference, SBP, Vitamin D, HBA1c, the status of hypertensive drugs, the anti-diabetic drugs, the type of lipid-lowering drug, duration of diabetes, creatinine and triglyceride concentration

^b^Model 2 was adjusted for height, SBP, Vitamin D, the status of hypertensive drugs, the anti-diabetic drugs, the type of lipid-lowering drug, duration of diabetes, and triglyceride concentration after dividing into different categories

SBP: systolic blood pressure, HDL-C: high density lipoprotein cholesterol

The RCS models illustrated that SBP below 130 mmHg (A), Vitamin D less than 20 mg/dL (B), height less than 160 cm (C), and triglyceride less than 150 mg/dL (D) concentrations were positively correlated with the probability of having HDL-C levels of more than 70 (Fig. 1). Each RCS had four knots based on the distribution of the associated variable. The reference values for the abovementioned curves were as follows: height of 160 cm, SBP of 130 mm Hg, triglyceride levels of 150 mg/dL, and vitamin D levels of 20 mg/dL.

## Discussion

The current cross-sectional, matched case–control study aimed to determine the correlates of high HDL-C levels. To date, many studies have been performed to evaluate the relationship between dyslipidemia and the risk of cardiovascular and diabetic complications; however, this study focused on individuals with high HDL levels and showed that having lower values of height, systolic blood pressure, triglycerides, and vitamin D increased the odds of having high HDL-C levels.

The current analysis demonstrated that there is no significant distinction in BMI and weight between individuals with high and low values of HDL-C as well as those with normal values of HDL-C. BMI and weight were greater in patients with both low and high HDL-C levels than in those with normal HDL-C levels. A cohort study in a Japanese population showed that women with a BMI ≥ 25.0 kg/m^2^ had a 1.54-fold greater risk of having low HDL-C concentrations than women with a BMI < 25.0 kg/m^2^ [36]. Ali, H.I., et al. reported that abdominal obesity and overweight were likely to increase the risk of having high total cholesterol, LDL-C, and TG and decrease HDL-C in adults [37]. Patients with high levels of HDL-C demonstrated a reduction in SBP as opposed to those with low and normal levels of HDL-C. K.-H. Cho et al. demonstrated that a decrease in HDL-C has the potential to increase SBP. This occurs as a result of increased binding of LDL to the scavenger receptor B-I (SR-BI) due to low levels of HDL-C. The SRB-I receptor in mitochondria produces aldosterone by signaling [38].

This analysis showed that individuals with high HDL-C (HDL-C levels of more than 70) had lower SBP. Actually, patients with SBP below 130 mm Hg had a lower risk of having high HDL-C levels than low and normal HDL-C levels. Based on the research conducted by Deng et al., elevated TC, LDL-C, and non-HDL-C can potentially lead to hypertension by elevating the levels of circulating endothelin-1. As a result, there was an inverse association between HDL-C and the incidence of hypertension [39]. Nakajima et al. reported an inverted J-shaped association between HDL-C and hypertension risk (≥ 140/90 mm Hg) in both sexes [40]. The present analysis also endorsed these findings. Furthermore, a different study discovered a correlation between higher DBP and SBP and lower levels of HDL-C [38]. HDL-C may contribute to a decreased angiotensin II response by reducing NAD(P)H oxidase activity and aortic angiotensin II type 1 receptor expression and also increasing endothelial NO synthase dimerization in contrast to LDL and oxidized LDL [41]. These findings were consistent with the current investigation that illustrated an inverse association between high HDL-C and blood pressure. Previous studies have shown that HDL-C was positively associated with hypertension due to the role of hypertension in the disturbance of HDL metabolism and the increased percentage of individuals with dysfunctional HDL-C. In hypertensive individuals, some HDL-C particles may not function to protect LDL from oxidation or control cholesterol efflux from the walls of blood vessels [42, 43]. However, Yang et al. proposed that HDL-C was not associated with systolic BP or hypertension in men [44].

The present survey discovered that the likelihood of having high HDL-C levels decreases as waist circumference and height increase. In addition, this analysis showed that the risk of having high HDL-C was higher in patients with heights less than 160 cm than in patients with heights more than 160 cm. Weschenfelder et al. demonstrated that serum triglyceride levels and waist circumference were significantly associated with lower HDL-C levels and smaller HDL-C particles in patients with heart failure [28]. Rosenbaum et al. reported that increasing waist circumference is associated with lower levels of large HDL particles (HDL2) and higher levels of small HDL particles (HDL3). In diabetes and cardiovascular disease, HDL3 has reduced anti-oxidative activity in patients with metabolic syndromes [45]. Williams et al. suggested that after one year, the weight loss resulted in a significant increase in HDL cholesterol, HDL2 cholesterol, and HDL2 mass[46]. Schekatolina et al. implied that Chylomicrons and VLDL were lipoprotein classes, rich in triglycerides, also known as TG-rich lipoproteins (TGRL). The metabolism of lipoprotein classes in the bloodstream is regulated through various pathways. The metabolism of HDL is specifically related to that of TGRL through the exchange of core lipids facilitated by the cholesteryl ester transfer protein (CETP), as well as through the transfer of surface fragments of TGRL to HDL that are generated during lipolysis by lipoprotein lipase (LPL). As a result, they showed a negative correlation between the concentrations of HDL-C and TG in circulation [47]. Miller and colleagues also demonstrated that a reduction of 50 mg/dl in triglyceride (TG) levels was associated with a 0.5 mg/dl increase in HDL-C levels in patients with TG levels of 200 mg/dl or higher. In addition, they found that the same reduction of TG levels resulted in a 1.7 mg/dl increase in HDL-C levels in individuals with TG levels below 200 mg/dl [48]. Another study showed that raising the plasma concentration of TG resulted in a greater decrease in HDL-C levels than in HDL-apolipoprotein A-I levels [49].

Navti et al. also suggested that increased BMI, waist circumference, and waist-to-height ratio were associated with lower HDL levels by increasing HDL-C catabolism due to insulin resistance, which suppresses lipolysis in an urban pediatric population in Cameroon [50]. Based on the current results, taller individuals exhibited decreased levels of HDL-C. Consistent with these results, Oh et al. implied that a decrease in sex hormones, an increase in insulin resistance, and damage to lecithin cholesterol acyl transferase activity could lead to an inverse relationship between height and HDL-C among patients with metabolic syndrome which could be attributed to the impact of aging on the metabolism of HDL [51]. A. M. Dattilo demonstrated that Subjects at a stabilized, reduced weight experienced a 0.009-mmol/L increase (P ≤ 0.01) in HDL-C per kilogram decrease in body weight. [52]. On the other hand, Shimizu et al. showed a significant positive correlation between height and HDL-C concentrations in individuals with BMI values greater than 25 kg/m^2^ [53]. A study also suggested that HDL-C was positively correlated with overweight in Korean adolescents and adults [54]. Freedman, D. S. et al. reported positive associations of BMI with TG and inverse associations with HDL-C in black and white children [55]. Another study suggested that BMI had a negative influence on HDL-C activity by prohibiting platelet accumulation and cholesterol efflux capacity and reducing large HDL-C subfractions. High HDL-C levels may enhance small HDL-C particles among the obese population [56].

A meta-analysis in 2021 demonstrated that vitamin D administration in postmenopausal women reduced HDL-C levels, especially when the HDL-C baseline value was more than 50 mg/dL in overweight women [57]. These findings align with the current results, which revealed that Vitamin D concentrations less than 30 ng/ml were associated with a decrease in the odds of having high HDL-C levels versus low HDL-C levels. Moreover, patients with vitamin D concentrations above 20 ng/ml were more likely to have HDL-C levels above 70 than those with normal HDL-C levels. Literature reported that 25(OH) vitamin D levels could improve lipid profiles. Furthermore, vitamin D was shown to have an inverse association with total cholesterol, low-density lipoprotein cholesterol, and triglycerides. However, how vitamin D impacts the lipid profile is not yet clear. Vitamin D may decrease cholesterol biosynthesis via increased 3-hydroxy-3-methylglutaryl-coenzyme A reductase (HMG-CoA) activity [58]. K. F. Faridi et. al. reported a positive association between vitamin D and more cardioprotective HDL-C particles via the role of vitamin D in reverse cholesterol transport. Furthermore, reverse cholesterol transport brings cholesterol out of lipid-laden macrophage sponge cells in atherosclerotic, plaques, such as HDL-C, for clearance from the circulation. Vitamin D deficiency could be related to impaired B-cell function which leads to insulin resistance, disruption of lipoprotein metabolism, increased TG levels, and decreased HDL cholesterol levels. Previous data have recommended that raising intestinal calcium absorption could lower the synthesis and secretion of hepatic TG. Therefore, vitamin D could arouse intestinal calcium absorption and prohibit TG synthesis and secretion. It has also been suggested that calcium could inhibit the intestinal absorption of fatty acids due to the formation of insoluble calcium–fatty acid complexes. Reduced absorption of fat, particularly saturated fatty acids, leads to reduced cholesterol concentrations in the serum. In addition, calcium could reduce the level of cholesterol by exciting the transformation of cholesterol into bile acids. Other studies have noted that vitamin D deficiency results in increased parathyroid hormone levels, which results in elevated TG and increased concentrations of vitamin D, decreasing serum PTH levels. This mechanism could influence TG concentrations [59–61]. Vitamin D supplementation could also decrease the HbA1c percentage by about 0.5% in T2DM patients. Therefore, a lack of vitamin D may cause dyslipidemia in elderly individuals with metabolic disorders [62, 63]. Research has shown that there is no significant impact of vitamin D supplementation on HDL levels in prediabetic individuals [64]. The traditional Japanese diet consists of a high intake of fish, miso, soy sauce, and vegetables; contributes to the prevention of ASCVD; lowers the risk for hypercholesterolemia, CAD, and non-HDL-C and increases serum HDL-C levels by genetic deficiency of cholesterol ester transfer protein (CETP) [65, 66].

These findings confirm the need for further investigation into the complexity of HDL to address impairments in HDL levels.

## Limitations

This study should be interpreted in light of its potential limitations. As this study is cross-sectional, it is not possible to determine the clinical impact of these results. Additionally, it is difficult to discern whether the relation between HDL-C and these variants is a true effect, a survivor effect, or a cohort effect. Therefore, it would be valuable to conduct cohorts to track the HDL-C levels in each patient with different variables. The study population was limited to individuals with T2D, which hinders the generalizability of the results. Second, potential confounding factors not obtained in the present study might have affected the findings. Since the study population was limited to the capital city, it is not possible to apply the results to a wider population. Hence, prospective longitudinal studies with larger sample sizes are recommended. Finally, the average age of about 60 years in the studied population and the coverage of the majority of the individuals by women could also limit the results of the study.

## Strengths

The key strength of the current survey was that three groups of HDL-C levels were matched by age and gender in this analysis. Considering the low frequency of high HDL-C in the population, a total of 170 patients with type 2 diabetes and HDL-C levels of more than 70 mg/dL were included in the current study. Until now, most research has focused on low levels of HDL and its complications; however, this study described the importance of focusing on high HDL levels.

## Conclusion

Due to recent findings that challenge the paradigm that high HDL-C levels are cardioprotective and because of a pattern of a U-shaped distribution of HDL-C, both low and high levels of HDL-C could be harmful. Therefore, strict control of the serum level of HDL-C may help in reducing the risk of cardiovascular events. This study focused on high HDL levels and the findings of the present study showed that SBP below 130 mm Hg, height less than 160 cm, Vitamin D less than 30 ng/ml, and lower triglyceride levels were positively associated with an increased likelihood of having high HDL-C levels. Further studies of high HDL-C levels are warranted to identify the causal role of HDL in health and disease states.

## Data Availability

The datasets used and/or analyzed during the current study are available from the corresponding author on reasonable request.

## Abbreviations

HDL-C: High-density lipoprotein cholesterol
T1D: Type 1 diabetes
ESRD: End-stage renal disease
SRB-1: Scavenger receptor BI proteins
LDL: Low-density lipoprotein
CETP: Cholesteryl ester transfer protein
CKD: Chronic kidney disease
OADs: Oral antidiabetic drugs
BMI: Body mass index
TG: Triglyceride
TC: Total cholesterol
FBG: Fasting blood glucose
2hPP: Two-hour postprandial glucose
HbA1c: Hemoglobin A1c
WC: Waist circumference
FBS: Fasting blood sugar
HPLC: High-performance liquid chromatography
AIP: Atherogenic index of plasma
VAI: Visceral adiposity index
CKD-EPI: Chronic Kidney Disease Epidemiology Collaboration
ADA: American Diabetes Association
NCEP ATP III: National Cholesterol Education Program-Adult Treatment Panel III
AHA: The American Heart Association
ACC: The American College of Cardiology
CI: Confidence interval
RCS: Restricted cubic spline
HMG-CoA: 3-Hydroxy-3-methylglutaryl-coenzyme A reductase
